# Prognostic factors among critically ill patients with community-acquired acute bacterial meningitis and acute kidney injury

**DOI:** 10.5935/0103-507X.20180030

**Published:** 2018

**Authors:** Sérgio Luiz Arruda Parente Filho, Livia Maria Barbosa Lima, Gilberto Loiola de Alencar Dantas, Débora de Almeida Silva, Victor de Matos Rolim, Antônio Mendes Ponte de Oliveira Filho, Iamê Tavares Vale e Melo, Geraldo Bezerra da Silva Junior, Elizabeth De Francesco Daher

**Affiliations:** 1 Programa de Pós-Graduação em Ciências Médicas, Departamento de Medicina Clínica, Hospital Universitário Walter Cantídio, Faculdade de Medicina, Universidade Federal do Ceará - Fortaleza (CE), Brasil.; 2 Programa de Pós-Graduação em Saúde Pública, Centro de Ciências da Saúde, Faculdade de Medicina, Universidade de Fortaleza - Fortaleza (CE), Brasil.

**Keywords:** Meningitis, Acute kidney injury, Prognosis, Mortality, Critical care

## Abstract

**Objective:**

To investigate prognostic factors among critically ill patients with community-acquired bacterial meningitis and acute kidney injury.

**Methods:**

A retrospective study including patients admitted to a tertiary infectious disease hospital in Fortaleza, Brazil diagnosed with community-acquired bacterial meningitis complicated with acute kidney injury. Factors associated with death, mechanical ventilation and use of vasopressors were investigated.

**Results:**

Forty-one patients were included, with a mean age of 41.6 ± 15.5 years; 56% were males. Mean time between intensive care unit admission and acute kidney injury diagnosis was 5.8 ± 10.6 days. Overall mortality was 53.7%. According to KDIGO criteria, 10 patients were classified as stage 1 (24.4%), 18 as stage 2 (43.9%) and 13 as stage 3 (31.7%). KDIGO 3 significantly increased mortality (OR = 6.67; 95%CI = 1.23 - 36.23; p = 0.028). Thrombocytopenia was not associated with higher mortality, but it was a risk factor for KDIGO 3 (OR = 5.67; 95%CI = 1.25 - 25.61; p = 0.024) and for mechanical ventilation (OR = 6.25; 95%CI = 1.33 - 29.37; p = 0.02). Patients who needed mechanical ventilation by 48 hours from acute kidney injury diagnosis had higher urea (44.6 *versus* 74mg/dL, p = 0.039) and sodium (138.6 *versus* 144.1mEq/L; p = 0.036).

**Conclusion:**

Mortality among critically ill patients with community-acquired bacterial meningitis and acute kidney injury is high. Acute kidney injury severity was associated with even higher mortality. Thrombocytopenia was associated with severer acute kidney injury. Higher urea was an earlier predictor of severer acute kidney injury than was creatinine.

## INTRODUCTION

Community-acquired bacterial meningitis (CABM) is a severe disease with a high rate of mortality that further increases if antibiotic therapy is delayed. Hence, this condition is considered a medical emergency.^(^^1^^,^^2^^)^ The main etiologic agents involved with CABM are *Streptococcus pneumoniae* and *Neisseria* meningitidis.^(^^3^^,^^4^^)^ *Haemophilus influenzae* was once an important pathogen of CABM in children. However, because of vaccines, *H. influenzae* is currently a rare cause of CABM. Moreover, because of widespread vaccination, CABM became more frequent in adults than in children.^(^^5^^)^ *Listeria monocytogenes* is another etiologic agent, but its role in CABM is usually restricted to extremes of age and immunocompromised patients.^(^^6^^)^

The classic triad of altered mental status, neck stiffness and fever is being slowly replaced by a tetrad that also includes headache. Although only a few patients present with all these classic findings, at least 2 of them will be present in 95% of cases.^(^^3^^)^ Definite diagnosis relies on cerebrospinal fluid (CSF) analysis, but no diagnostic procedure should prevent early treatment of suspected cases.

Overall mortality in CABM ranges from 8.5 to 25%.^(^^7^^-^^10^^)^ Patients may require intensive care, mostly due to severe impairment of mental status, septic shock and organ failure.^(^^10^^)^ In this group of patients, mortality rises to 40 - 56%.^(^^11^^,^^12^^)^ Among septic patients, mortality can be as high as 77.4%.^(^^13^^)^

Acute kidney injury (AKI) has a significant prevalence (6 - 23%) in intensive care units (ICU)^(^^14^^,^^15^^)^ and is also an important determinant of outcome in this group of patients.^(^^16^^,^^17^^)^ Nevertheless, there are very few studies assessing AKI in critically ill patients with meningitis. The aim of this study was to investigate predictors of poorer outcome in critically ill patients with CABM and AKI.

## METHODS

This is a retrospective study including patients admitted to the ICU of a tertiary care infectious disease hospital in Fortaleza (CE), Brazil with a confirmed diagnosis of CABM complicated with AKI in the period between January 2003 and December 2015. This study was reviewed and approved by the Ethics Committee of *Hospital São José* de *Doenças Infecciosas* (CAAE: 12565613.4.0000.5054).

Community-acquired bacterial meningitis was diagnosed by CSF analysis. Patients who presented with previous kidney disease or used nephrotoxic drugs that were not related to the current hospitalization were excluded from the study. Acute kidney injury was diagnosed and classified according to Kidney Disease: Improving Global Outcomes (KDIGO) criteria.^(^^18^^)^ To determine the impact of CABM infection on the kidney, renal function was assessed using serum creatinine and urea as well as urine output quantification. Further data included patient demographics, drugs in use, and in-hospital survival. Hemoglobin, hematocrit, white blood cell count, platelet count, sodium, potassium, aspartate aminotransferase (AST), alanine aminotransferase (ALT) and arterial blood gas analysis were among the laboratory data collected in the study.

Clinical and laboratory data were compared between groups, such as survivors *versus* non-survivors, severe *versus* non-severe AKI (KDIGO 3 *versus* non-KDIGO 3), vasopressors use *versus* non-vasopressors use, mechanical ventilation (MV) *versus* non-MV. Parameters on admission and at AKI diagnosis were also correlated with poor outcome in the following 48 hours.

Statistical analysis was performed using Statistical Package for Social Science (SPSS), version 20.0 for Windows (IBM, USA). The Kolmogorov-Smirnov test was applied to sort out the continuous variables that follow a normal distribution from the variables that do not follow a Gaussian curve. Student's *t* or Mann-Whitney tests were applied accordingly. Likewise, chi-squared or Fisher's exact tests were performed to make comparisons between categorical variables of different groups of patients. Variables that presented a relationship in the chi-squared or Fisher's exact tests (p < 0.05) underwent multivariate analysis by binary logistic regression. Continuous variables were recoded into categorical variables so that chi-squared or Fisher's exact tests could be performed; if statistical significance was found, binary logistic regression was carried out. Significance was set on 5% (p < 0.05). For the assessment of mortality outcome, the variables entered in the logistic regression analysis were "need for vasopressors" and "KDIGO 3"; for respiratory outcome, the variables "thrombocytopenia" and "sodium levels above 140mEq/L" were analyzed; for renal outcome, "thrombocytopenia" was entered into regression analysis; and for circulatory outcome (need for vasopressors), the variable "hematocrit above 35%" was analyzed.

## RESULTS

A total of 41 patients were included, with a mean age of 41.6 ± 15.5 years, and 56% were males. Mean time between ICU admission and AKI diagnosis was 5.8 ± 10.6 days. Overall mortality was 53.7%. There was no statistically significant difference between survivors and non-survivors regarding age (42.72 ± 19.09 *versus* 41.76 ± 11.84 years, p = 0.855). The most common comorbidity was diabetes mellitus (22%), followed by arterial hypertension (14.6%) and liver disease (7.3%). One patient had history of cancer (2.4%).

Non-survivors required vasopressors during ICU stay more often than survivors (p = 0.001) and, in the multivariate analysis, need for vasopressors was a risk factor for dying (p = 0.004; OR = 12.5, 95%CI = 2.23 - 70.19). However, patients who used vasopressor drugs in the first 48 hours after AKI diagnosis did not present increased mortality (p = 0.220).

Vancomycin was administered to 24 patients (58.5%), ceftriaxone to 32 patients (78%), furosemide to 9 patients (22%), polymyxin B to 6 patients (14.6%) and aminoglycosides to 4 patients (9.8%). Twenty-nine patients (70.7%) required vasopressors. Vancomycin (p = 0.678), ceftriaxone (p = 0.113), polymyxin B (p = 0.645) and aminoglycosides (p = 1.00) did not correlate with severer AKI (KDIGO 3). None of these drugs was associated with higher or lower mortality.

According to KDIGO criteria, 10 patients were classified as stage 1 (24.4%), 18 as stage 2 (43.9%) and 13 as stage 3 (31.7%). KDIGO stage 3 was associated with increased mortality (p = 0.018) but not with increased need for vasopressors (p = 0.276) or invasive ventilation (p = 0.458). In the multivariate analysis, AKI classification in KDIGO 3 significantly increased the risk of death (p = 0.028; OR = 6.67, 95%CI = 1.23 - 36.23). There was no statistically significant age difference between patients who developed KDIGO 3 and those who did not (43.08 ± 13.51 *versus* 41 ± 16.66 years, p = 0.698). Seven patients required hemodialysis (17.1%).

Patients who developed early AKI (in the first 24 hours after ICU admission) did not have higher mortality (p = 1.00) but required MV in the first 2 days after AKI diagnosis more often than those who had delayed AKI (p = 0.029). Likewise, patients who needed MV by 48 hours from AKI diagnosis had greater urea (44.62 ± 27.30 *versus* 74.05 ± 33.69mg/dL, p = 0.039) and sodium levels (138.62 ± 3.50 *versus* 144.11 ± 6.54mEq/L, p = 0.036) and presented a tendency to higher AST levels (50 ± 44.62 *versus* 330.82 ± 754.61UI/L, p = 0.052). Urea levels above 85mg/dL at AKI diagnosis tended to predict the need for MV in 48 hours (p = 0.061). In the multivariate analysis, sodium levels above 140mEq/L at the time of AKI diagnosis showed a tendency to increase the risk of requiring MV in 48 hours (p = 0.061; OR = 6, 95%CI = 0.92 - 39.18). There was no statistically significant age difference between patients who required intubation and those who did not (41.67 ± 14.54 *versus* 41.7 ± 19.19 years, p = 0.995).

Tables 1 - 4 display mean laboratory values on ICU admission and AKI diagnosis and correlate them with worse outcomes. Patients who developed KDIGO 3 AKI had higher sodium levels on ICU admission, as shown in table 2. At AKI diagnosis, the only parameter apart from creatinine and urea that was significantly different in the KDIGO 3 group was platelet count, as shown in table 4. However, patients who developed thrombocytopenia (platelet count < 150.000/mm^3^) during the ICU stay did not have increased mortality (p = 1.00).

**Table 1:** Comparison of mean laboratory parameters on intensive care unit admission of patients who required and those who did not require invasive mechanical ventilation or vasopressors

Parameters on ICU admission	Invasive ventilation in 48 hours	No invasive ventilation in 48 hours	p value	Vasopressors	No vasopressors	p value
Creatinine (mg/dL)	1.54 ± 1.30	0.63 ± 0.16	0.006*	1.45 ± 1.3	0.76 ± 0.4	0.263
Urea (mg/dL)	56 ± 32	29 ± 10.8	0.004*	51.1 ± 32.7	40.8 ± 20.6	0.479
Hemoglobin (g/dL)	12.2 ± 1.8	11.5 ± 1.6	0.443	12.1 ± 1.8	11.4 ± 1.3	0.455
Platelets (10^5^/mm^3^)	21.2 ± 19	22.7 ± 7.9	0.868	21.1 ± 17.7	23.8 ± 8.3	0.770
AST (IU/L)	85 ± 10.3	25.2 ± 10.6	0.074	72.1 ± 98.2	37.5 ± 33.7	0.878
ALT (IU/L)	67.4 ± 62.6	27 ± 27.7	0.101	57.3 ± 48.8	70.3 ± 101.2	0.846
Sodium (mEq/L)	143.4 ± 5.1	138.2 ± 3.0	0.046*	142.9 ± 4.8	138.5 ± 4.4	0.113
Potassium (mE/L)	3.6 ± 0.8	3.2 ± 0.7	0.433	3.4 ± 0.9	3.6 ± 0.2	0.568

ICU - intensive care unit; AST - aspartate aminotransferase; ALT - alanine aminotransferase. Student’s *t* test.

* p values ≤ 0.05 were considered statistically significant. Values expressed as mean ± standard deviation.

**Table 2:** Comparison of mean laboratory parameters on intensive care unit admission of patients who died/survived and developed/did not develop Kidney Disease: Improving Global Outcomes (KDIGO) stage 3 acute kidney injury

Parameters on ICU admission	Non-survivors	Survivors	p value	KDIGO Stage 3	Non-KDIGO Stage 3	p value
Creatinine (mg/dL)	1.18 ± 0.98	1.18 ± 1.17	1.000	2.43 ± 1.83	0.89 ± 0.49	0.116
Urea (mg/dL)	45.69 ± 31.36	50.31 ± 31.2	0.710	77.28 ± 36.91	38.8 ± 20.76	0.002*
Hemoglobin (g/dL)	12.11 ± 2.1	11.79 ± 1.38	0.692	13.25 ± 1.34	11.80 ± 1.74	0.276
Platelets (10^5^/mm^3^)	24.49 ± 20.95	18.78 ± 9.58	0.443	16.05 ± 5.59	22.25 ± 16.87	0.619
AST (IU/L)	63.75 ± 108.07	64.90 ± 75.13	0.979	168 ± 227.69	51.44 ± 61.41	1.000
ALT (IU/L)	50.75 ± 48.91	67.44 ± 65.07	0.563	71 ± 91.92	58.07 ± 55.46	0.773
Sodium (mEq/L)	142.4 ± 4.99	141.7 ± 5.21	0.763	148.5 ± 0.71	141.33 ± 4.71	0.050*
Potassium (mEq/L)	3.43 ± 0.67	3.49 ± 0.75	0.871	4.15 ± 1.63	3.38 ± 0.69	0.201

ICU - intensive care unit; AST- aspartate aminotransferase; ALT- alanine aminotransferase. Student’s *t* test.

* p values ≤ 0.05 were considered statistically significant. Values expressed as mean ± standard deviation.

**Table 3:** Comparison of mean laboratory parameters at acute kidney injury diagnosis of patients who required and those who did not require invasive ventilation or vasopressors

Parameters at AKI diagnosis	Invasive ventilation in 48 hours	No invasive ventilation in 48 hours	p value	Vasopressors	No vasopressors	p value
Creatinine (mg/dL)	1.99 ± 1.25	1.47 ± 0.83	0.216	2.02 ± 1.1	1.57 ± 0.53	0.093
Urea (mg/dL)	74.05 ± 33.69	44.62 ± 27.30	0.039*	79.11 ± 41.82	60.17 ± 37.37	0.186
Hemoglobin (g/dL)	11.34 ± 2.2	11.65 ± 2.02	0.734	12.05 ± 2.27	10.92 ± 1.93	0.139
Platelets (10^5^/mm^3^)	14.44 ± 13.04	20.20 ± 12.27	0.294	14.64 ± 11.74	18.12 ± 12.2	0.400
AST (IU/L)	330.81 ± 754.61	50 ± 44.62	0.428	242.5 ± 631.15	39 ± 28.66	0.910
ALT (IU/L)	177.82 ± 333.03	59.4 ± 42.83	0.450	129.56 ± 279.89	66.6 ± 72.93	0.569
Sodium (mEq/L)	144.11 ± 6.54	138.62 ± 3.5	0.036*	145.35 ± 9.19	142 ± 8.79	0.297
Potassium (mEq/L)	4.06 ± 1.41	3.52 ± 0.84	0.333	3.63 ± 1.13	4.28 ± 1.09	0.105

AKI - acute kidney injury; AST - aspartate aminotransferase; ALT - alanine aminotransferase. Student’s *t* test.

* p values ≤ 0.05 were considered statistically significant. Values expressed as mean ± standard deviation.

**Table 4:** Comparison of mean laboratory parameters at acute kidney injury diagnosis of patients who died/survived and developed/did not develop Kidney Disease: Improving Global Outcomes (KDIGO) stage 3 acute kidney injury

Parameter at AKI diagnosis	Non-survivors	Survivors	p value	KDIGO Stage 3	Non-KDIGO Stage 3	p value
Creatinine (mg/dL)	2.03 ± 0.89	1.59 ± 0.91	0.136	2.87 ± 0.94	1.41 ± 0.56	<0.001*
Urea (mg/dL)	80.3 ± 44.67	65.72 ± 37.47	0.286	100.92 ± 30.78	59.46 ± 38.75	0.002*
Hemoglobin (g/dL)	11.91 ± 2.74	11.61 ± 1.41	0.661	11.84 ± 3.09	11.65 ± 1.71	0.806
Platelets (10^5^/mm^3^)	13.58 ± 13.04	17.98 ± 10.47	0.257	9.85 ± 7.99	18.49 ± 12.47	0.029*
AST (IU/L)	308.6 ± 804.98	80.8 ± 70.46	1.000	493.33 ± 1033.03	74.33 ± 58.68	0.910
ALT (IU/L)	156.6 ± 357.07	76.3 ± 57.92	0.631	227 ± 462.47	69.6 ± 50.61	0.569
Sodium (mEq/L)	145.65 ± 10.4	142.78 ± 7.34	0.337	145.67 ± 11.99	143.65 ± 7.58	0.533
Potassium (mEq/L)	3.83 ± 1.35	3.85 ± 0.91	0.955	4.32 ± 1.53	3.62 ± 0.87	0.161

AKI - acute kidney injury; AST - aspartate aminotransferase; ALT - alanine aminotransferase. Student’s *t* test.

* p values ≤ 0.05 were considered statistically significant. Values expressed as mean ± standard deviation.

At the time of AKI diagnosis, thrombocytopenia was associated with a higher incidence of KDIGO 3 (p = 0.018) and need for dialysis (p = 0.044). In the multivariate analysis, it was a risk factor for KDIGO 3 (OR = 5.67, 95%CI = 1.25 - 25.61; p = 0.024) and tended to increase the risk of requiring dialysis (OR = 8.77, 95%CI = 0.94 - 81.67; p = 0.057). Thrombocytopenia at any point of the ICU stay was a risk factor for invasive ventilatory support (OR = 6.25, 95%CI = 1.33 - 29.37; p = 0.02) in the multivariate analysis.

Hematocrit above 35% at AKI diagnosis correlated with a higher need for vasopressors (p = 0.041) and an increased risk for needing vasopressors in the multivariate analysis (p = 0.036; OR = 4.75, 95%CI = 1.11 - 20.39). There was no statistically significant age difference between patients who required such drugs and those who did not (41.9 ± 13.1 *versus* 41 ± 20.8 years, p = 0.860).

## DISCUSSION

Acute kidney injury is still a poorly studied complication among patients with CABM, a disease that was associated in the present study with very high mortality at a rate higher than described in the literature.^(^^11^^,^^12^^)^ A study performed in another city in Brazil, which included 294 CABM patients from both the ICU and general wards, highlighted a threefold increase in mortality among patients with thrombocytopenia or a positive blood culture.^(^^19^^)^ Low platelet count also increases mortality in septic patients.^(^^20^^)^ Thrombocytopenia was an important prognostic factor in this study , but it was not a predictor of mortality. This may be explained by the obvious differences between the samples of the two studies. Nonetheless, the fact that thrombocytopenia was a risk factor for intubation and, at AKI diagnosis, for severer AKI and dialysis corroborates that the clinician should be careful to monitor variations in platelet count. A study by Hollestelle et al. has suggested that disseminated intravascular coagulation and consumption of von Willebrand factor and granzyme-B may be involved in the mechanism of thrombocytopenia in CABM.^(^^21^^)^

Thrombocytopenia has been associated with severe AKI in various conditions, such as heat stroke, Hantaan virus infection, and other infectious diseases, including leptospirosis, dengue and malaria.^(^^22^^-^^24^^)^ The mechanism of such an association is still elusive. The present study suggests that CABM follows the same pattern, since thrombocytopenia at AKI diagnosis increased almost sixfold the risk of KDIGO 3 and tended to be a risk factor for dialysis.

Higher serum creatinine correlated with worse renal outcome only at AKI diagnosis, while higher serum urea predicted KDIGO 3 both on admission and AKI diagnosis. Since KDIGO diagnostic criteria for AKI diagnosis are based on creatinine rather than urea levels, it is expected that AKI is diagnosed with the rise of creatinine in the serum. However, table 2 shows that patients who eventually developed KDIGO 3 AKI already had higher urea levels on ICU admission before the rise in serum creatinine took place. This finding suggests a faster increase in urea than in creatinine levels.

Another study compared early and delayed AKI in the same ICU, but in a different period from the present study. It included 147 patients with various infectious diseases who developed AKI and drew the conclusion that delayed AKI predicted MV and showed a tendency to increase mortality, although patients who developed early AKI had higher APACHE II scores.^(^^25^^)^ In the present study, such a tendency was not observed, and early AKI was associated with intubation within 48 hours from AKI diagnosis.

Older age was not a predictor of severe AKI, death or need for vasopressors or invasive ventilatory support. However, a study that included 65 CABM critically ill patients (who did or did not develop AKI) showed that patients who presented "adverse clinical outcomes" (e.g., neurologic sequelae or death) were older than those who did not.^(^^11^^)^

*S. pneumoniae* is capable of elevating vascular endothelial growth factor (VEGF) levels in CSF. It could be involved in the pathogenesis of brain edema in some CABM patients via an increase in vascular permeability, although VEGF inhibition did not diminish cerebral edema.^(^^26^^,^^27^^)^ However, VEGF production stimulation is not limited to the CSF, as it was first demonstrated that *S. pneumoniae* was capable of inducing VEGF production by human neutrophils in the peripheral blood.^(^^28^^)^ This phenomenon may constitute a mechanism of hypotension via increased vascular permeability and third-space fluid loss. Such a proposition is consistent with the finding that higher hematocrit at AKI diagnosis elevated the risk of requiring vasopressors almost fivefold.

Some laboratory variables at AKI diagnosis seemed to predict intubation in 48 hours, such as higher urea and sodium levels. There are studies in the literature proposing an association between high urea levels and an increased need for ventilatory support.^(^^29^^,^^30^^)^ A recent prospective study has pointed to urea levels > 49.25mg/dL as an independent risk factor for re-intubation in a surgical ICU.^(^^27^^)^ Clark and Lettieri^(^^30^^)^ developed a very specific clinical model to predict prolonged intubation, in which urea > 25mg/dL increased the risk of requiring MV for more than 14 days. A previous study by Milhaud et al.^(^^12^^)^ also showed that high urea is an important prognosis factor in CABM patients.

The main limitations of this study derive from its retrospective nature and small sample size. Data regarding the etiological diagnosis of the agents causing CABG were not available. The study was conducted in only one region of Brazil, so disease patterns may be different in other regions of the globe.

## CONCLUSION

Mortality among critically ill patients with community-acquired bacterial meningitis complicated with acute kidney injury is very high, and the clinician should be particularly careful to monitor prognostic factors in order to improve patient care. High hematocrit, serum urea and thrombocytopenia were associated with worse hemodynamic, ventilatory and renal outcomes, respectively. Low sodium levels at acute kidney injury diagnosis may also be an important prognostic factor in this group of patients. Higher serum urea was an earlier predictor of worse renal outcome compared to serum creatinine.
